# Designing multiple degenerate primers via consecutive pairwise alignments

**DOI:** 10.1186/1471-2105-9-55

**Published:** 2008-01-27

**Authors:** Hamed Shateri Najafabadi, Noorossadat Torabi, Mahmood Chamankhah

**Affiliations:** 1 Department of Biotechnology, University of Tehran, Enghelab Ave., Tehran, Iran; 2 Institute of Parasitology, McGill University, 21,111 Lakeshore Road, Quebec H9X 3V9, Canada; 3 Department of Molecular Biology, Princeton University, One Clio Hall, Princeton, NJ 08544, USA; 4 Nanobiotechnology Research Center, Avesina Research Institute, Shahid Beheshti University, Evin Ave., Tehran, Iran

## Abstract

**Background:**

Different algorithms have been proposed to solve various versions of degenerate primer design problem. For one of the most general cases, multiple degenerate primer design problem, very few algorithms exist, none of them satisfying the criterion of designing low number of primers that cover high number of sequences. Besides, the present algorithms require high computation capacity and running time.

**Results:**

PAMPS, the method presented in this work, usually results in a 30% reduction in the number of degenerate primers required to cover all sequences, compared to the previous algorithms. In addition, PAMPS runs up to 3500 times faster.

**Conclusion:**

Due to small running time, using PAMPS allows designing degenerate primers for huge numbers of sequences. In addition, it results in fewer primers which reduces the synthesis costs and improves the amplification sensitivity.

## Background

Polymerase Chain Reaction, or PCR [1], is a ubiquitous technique which amplifies a specific region of DNA, so that enough copies of that region is available to be adequately tested, sequenced or manipulated in other fashions. In order to use PCR, one must know the exact sequences which lie on either side of the DNA region of interest. These sequences are used to design two synthetic DNA oligonucleotides, or *primers*, one complementary to each strand of the DNA double-helix and lying on opposite sides of the target region. The primers are typically 20–30 nucleotides long.

**Figure 1:**
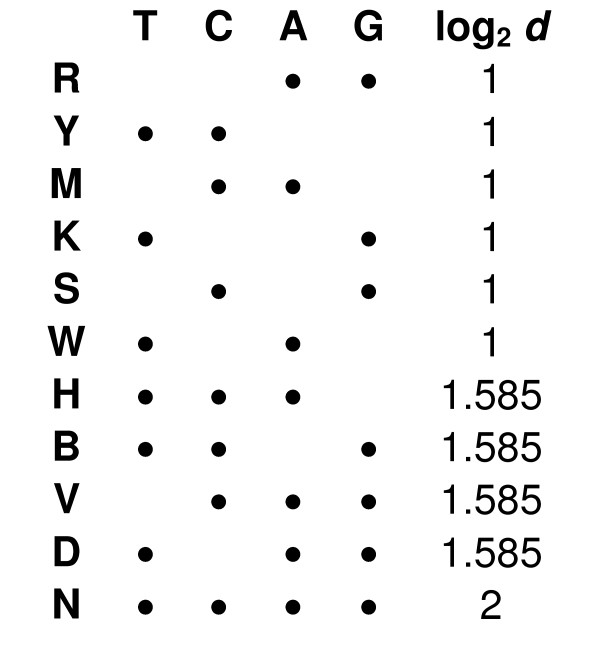
IUPAC nomenclature of mixed bases [13]. The base-2 logarithm of degeneracy of each mixed base is also represented.

Assuming ∑ = {T, C, A, G} is the DNA alphabet [2], a sequence (e.g. a primer) can be shown as *S *= *x*_1_*x*_2_...*x*_*l*_, where *x*_*i *_⊆ ∑, *x*_*i *_≠ Ø and *l *is the length of *S*. A sequence is called *degenerate *if some of its positions have several possible bases [3]. For example, in the primer P* = {G}{G}{C,G}{A}{T,C,G}{A} the third position is C or G and the fifth is C, T or G. The IUPAC illustration of P* will be GGSABA (Figure 1). The *degeneracy *of a sequence is the number of unique sequence combinations it contains, which can be calculated as *d*(*S*) = Π^*l*^_*i*=1_|*x*_*i*_|. For example, *d*(P*) = 1 × 1 × 2 × 1 × 3 × 1 = 6. Degenerate primers are useful for amplifying several related genomic or cDNA sequences, and have been exploited in various applications such as amplifying DNA sequences of homologous genes or genes from a particular protein family and analysis of species diversity [4-6].

Traditionally, degenerate primers were designed manually by examining multiple alignments of the target sequences. However, several programs are now available for designing degenerate primers for aligned sequences. CODEHOP [7] and DePiCt [8] are programs for designing degenerate primers for aligned protein sequences in order to identify new members of protein families. For each given multiple sequence alignment, CODEHOP constructs a pair of primers. Each primer consists of a degenerate 3' core region, typically with degeneracy of at most 128, and a 5' consensus sequence that stabilizes annealing. It works well for small sets of proteins, taking into account the codon usage of the target genome as well as the desired annealing temperature. However, it is inappropriate for constructing primers with high degeneracy on large sets of long genomic sequences. DePiCt clusters the sequences using a simple similarity score and then designs a pair of primers for each cluster by translating conserved blocks of amino acids into nucleotides.

In order to obtain primers that cover a large number of known genes and thus have a good chance to detect new related ones, one should obviously use highly degenerate primers (the primer *P *= *p*_1_*p*_2_...*p*_*l *_covers the sequence *S *if there is a substring *F *of length *l *in *S *where for each character *f*_*i *_in *F*, *f*_*i *_⊆ *p*_*i*_). On the other hand, in order to reduce the probability of amplifying unrelated sequences, the degeneracy must be bounded. This contradictory nature of the degenerate primer design (DPD) problem has led to definition of several variants of this problem, all of which are NP-complete:

1. Maximum Coverage Degenerate Primer Design (MC-DPD) tries to find a primer of length *l *and degeneracy at most *d*_*max *_that covers a maximum number of strings (sequences) of a given input set, each of length *l*. HYDEN [9], an algorithm based on a heuristic approach, basically addresses this variant of DPD problem and was first used to design degenerate primers for a set of genomic sequences in order to find new human olfactory receptor genes [9,10].

2. Minimum Degeneracy Degenerate Primer Design (MD-DPD) addresses the problem of finding a primer of length *l *and minimum degeneracy that covers all the input strings, each of which having a length equal to or greater than *l*.

3. Minimum Primers Degenerate Primer Design (MP-DPD) is applied when a set of strings of length *l *is given, and finds a minimum number of primers of length *l *and degeneracy at most *d*_*max*_, so that each input string is covered by at least one primer.

MP-DPD has the constraint that all input sequences are of the same length as the primers, which is not the case for most real situations. Removing this constraint, i.e. allowing the strings to have arbitrary lengths, results in a more general problem, Multiple Degenerate Primer Design (MDPD) [2]. MDPD is to find a minimum number of primers of length at least *l*_*min *_and degeneracy at most *d*_*max*_, given a set of *n *strings of various lengths (equal to or greater than *l*_*min*_), so that each input string is covered by at least one primer. A currently available algorithm for designing multiple degenerate primers, called PT-MIPS [2], has been developed in the context of SNP genotyping. It uses an iterative beam-search technique to construct progressively a set of primers until all sequences are covered.

In this work, we introduce a new algorithm for solving MDPD problems which consecutively uses an *ad hoc *pairwise alignment for multiple primer selection – hence called PAMPS. We will show that PAMPS performs better than previous algorithms on different sets of input strings, i.e. results in smaller number of primers in a considerably less computation time.

## Results and Discussion

**Figure 2:**
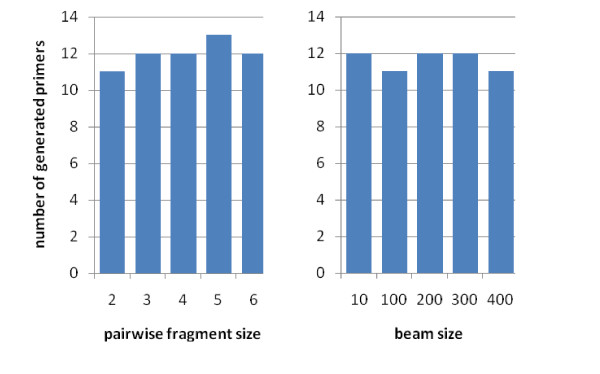
Performance of PT-MIPS [2] for different input parameters. **(left) **beam size = 10, variable pairwise fragment size; **(right) **pairwise fragment size = 6, variable beam size. A set of 40 random sequences each of length50 is used to generate primers each of length 15 and maximum degeneracy of 10^4^. Increasing the beam size or decreasing the pairwise fragment size improves the algorithm performance slightly, but increases the computation time significantly, making large analyses like that of Figure 3 impossible.

To compare the performance of PAMPS with PT-MIPS (Souvenir et al., 2003), different sets of random sequences were generated. Each set contained 20–100 sequences with similar length, but the lengths of sequences varied among different sets; sequences were of lengths 15–50 nucleotides. For each number of sequences and each sequence length three random sets were generated and the results were averaged over each triplet. PT-MIPS asks the user for "beam size" as well as "pairwise fragment size" (for more discussion, see [2]). As changing the values of these parameters did not improve the results of PT-MIPS significantly (Figure 2), we used the default values of PT-MIPS, 10 and 6, for beam size and pairwise fragment size, respectively.

**Figure 3:**
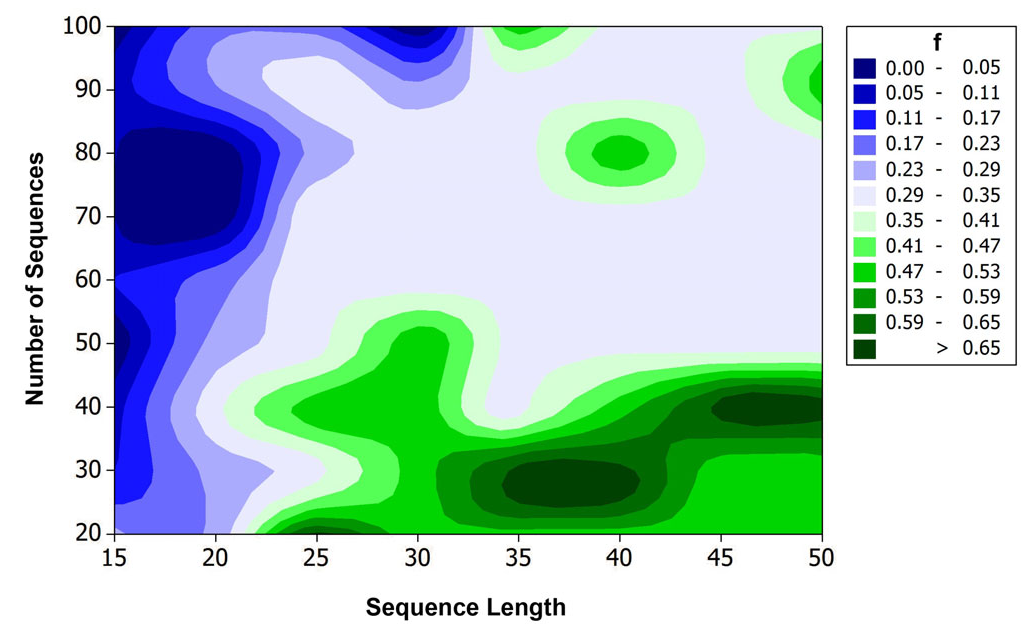
Efficiency of PAMPS (*f*) compared to PT-MIPS. *f *is defined as (*m*_mips_-*m*_pamps_)/*m*_mips _where *m*_mips _is the number of primers produced by PT-MIPS and *m*_pamps _represents the number of primers produced by PAMPS. Multiple sets of different numbers of random sequences with varying lengths are used to compare PT-MIPS and PAMPS. Each set of sequence is once used as input of PT-MIPS and once as input of PAMPS. Minimum primer length was set as 15 and maximum degeneracy as 10^4^.

**Figure 4:**
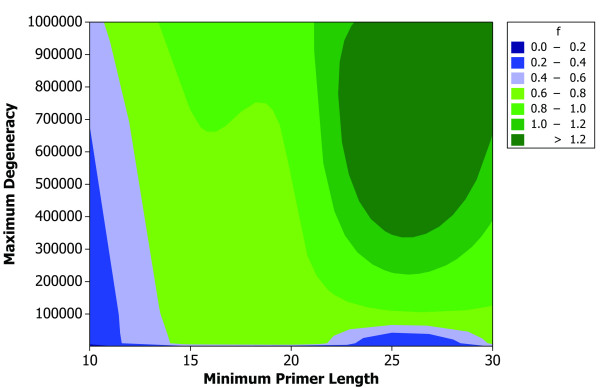
Contour plot of *f *for different primer lengths and degeneracy values. A set of 40 random input sequences each of length 50 is used to compare PT-MIPS and PAMPS, requesting these algorithms to generate a range of primer lengths as well as maximum degeneracy values.

Both PAMPS and PT-MIPS were used to solve MDPD problem for each of the above mentioned random sets given *l*_*min *_= 15 and *d*_*max *_= 10^4^. Almost always PAMPS resulted in smaller primer sets. Only in few cases both PAMPS and PT-MIPS produced primer sets with equal sizes. To compare PAMPS and PT-MIPS quantitatively, we defined efficiency of PAMPS as

(2) $$f_{PAMPS}=\frac{m_{MIPS}-m_{PAMPS}}{m_{MIPS}},$$

where *m*_*MIPS *_and *m*_*PAMPS *_represent the number of primers designed by PT-MIPS and PAMPS, respectively. Figure 3 illustrates the values of *f*_*PAMPS *_for different numbers of sequences and different sequence lengths. Obviously PAMPS outperforms PT-MIPS, especially when smaller sets of sequences or long sequences are used. In most situations, PAMPS decreases the number of final primers by 30%–35%. PAMPS outperforms PT-MIPS for a wide range of primer sizes and maximum degeneracy values (Figure 4).

**Figure 5:**
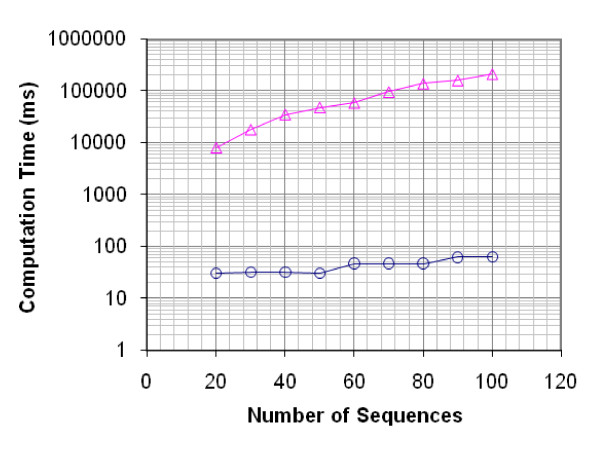
Comparison of PAMPS (open circles) and PT-MIPS (open triangles) in terms of compuatation time. Different numbers of sequences with length 50 nt are used to generate primers of length at least 15 nt and degeneracy at most 10^4^. In the case of 100 sequences, the run time for PT-MIPS is 213s, while for PAMPS it is 63 ms which is 3380 times faster.

Comparing the run time of PAMPS and PT-MIPS shows that PAMPS is astonishingly faster than PT-MIPS (both software were run on a 2.4 GHz Intel^® ^CPU): solving MDPD problem for 100 input sequences of length 50 nucleotides is about 3380 times faster using PAMPS compared to PT-MIPS (Figure 5). This allows PAMPS to be used to design highly degenerate primers for thousands of input sequences each hundreds of nucleotides long. Hence, even though the number of designed primers using PAMPS and PT-MIPS may converge as the number of input sequences increases, considering computation time strongly encourages using PAMPS; for an input set of 10^4 ^random sequences of length 2000, PAMPS needs an average time of 228 seconds to complete the computations on a 2.0 GHz Intel^® ^Core™ 2 CPU. We should mention that PT-MIPS did not yield in any results after three days of running the same job as PAMPS. Based on previous comparisons of PAMPS and PT-MIPS, we can estimate that for PT-MIPS it takes more than nine days to finish a job like this.

PT-MIPS [2] is previously compared with HYDEN [9]. Though HYDEN is basically designed to solve MC-DPD problems, it can be used iteratively to approximate MDPD problems, i.e. once a primer of length *l*_*min *_and degeneracy at most *d*_*max *_is found that covers the maximum number of input sequences, the sequences which are covered by this primer are subtracted from input set and HYDEN is run again on the remaining sequences. By repeating this procedure, eventually a set of primers is obtained which covers all sequences. Since it has been shown that PT-MIPS outperforms HYDEN [2] and as PAMPS outperforms PT-MIPS, we avoided the direct comparison of PAMPS and HYDEN.

**Figure 6:**
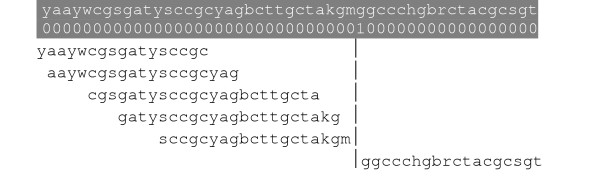
Output of PAMPS for a set of 20 sequences, given *l*_*min *_= 17 and *d*_*max *_= 64 (gray background). In this case the output is a single sequence which is split just before a G nucleotide that is marked by 1 underneath it. Each nucleotide belongs to at least a sub-sequence of length at least 17 and degeneracy at most 64 which does not pass over the split point (the split point is indicated by the vertical dashed line; see Methods for description of split point). All sub-sequences that meet these criteria are indicated below the output. Obviously not all sub-sequences of length 17 nt have degeneracy less than 64. The possibility of choosing between several sub-sequences allows the user to design more compatible pairs of primers, e.g. primers with close annealing temperatures.

The output of PAMPS is a list of primers, most of which are longer than *l*_*min *_(Figure 6). Therefore, any subsequences of length *l*_*min *_from each output can be selected to be used for PCR amplification. If the longest possible PCR product is desired, then the very upstream subsequence should be used. However, for most PCR reactions it is important to have primers with similar annealing temperatures if a mixture of primers is used. Since different combinations of primers can be chosen, it is possible to select the primers that have similar annealing temperatures. PAMPS is accompanied by a simple iterative algorithm provided in a separate software that chooses the best combination of primers in order to achieve the minimum variance among primer annealing temperatures. Primer annealing temperatures are estimated as [11].

## Conclusion

In this work we presented a new algorithm, called PAMPS, for solving MDPD problems. PAMPS exploits an altered pairwise alignment to select the subsequences which may be merged into degenerate primers. PAMPS was shown to run significantly faster than a previously developed software, PT-MIPS [2] and also gives better results (i.e. smaller sets of primers), reducing the synthesis costs of primers. Besides, when the number of mixed primers that are used in a PCR reaction are decreased, the concentration of the reacting primer increases, which usually improves the sensitivity of amplification. PAMPS, in contrast to previous algorithms, does not restrict the output to the exact primer length that was given; instead, it may result in primers longer than the requested length which allows selecting an appropriate primer in terms of annealing temperature. PAMPS can be used to design degenerate primers for amplification of genes with uncertain sequences, such as new members of gene families or libraries of antibody variable fragments. An implementation of PAMPS is provided in the Additional file 1.

## Methods

### Merging two aligned sequences

**Figure 7:**
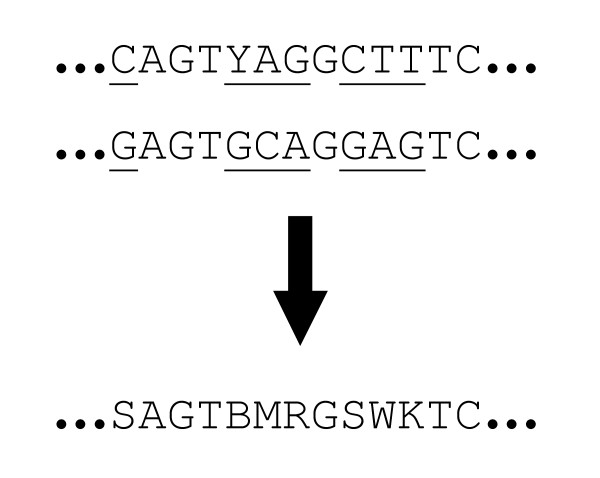
An example of merging two non-gapped aligned sequences into a single sequence. Bases that differ in the two sequences are underlined.

Assume that the alignment of two sequences is given. Merging two non-gapped aligned sequences *S*_1 _= *x*_1_*x*_2_...*x*_*l *_and *S*_2 _= *y*_1_*y*_2_...*y*_*l *_results in *S*_1,2 _= (*x*_1 _∪ *y*_1_)(*x*_2 _∪ *y*_2_)...(*x*_*l *_∪ *y*_*l*_) (Figure 7). Obviously, the regions of each sequence that are located in a gap are of no value in designing a degenerate primer that can cover both sequences. Therefore, these regions should be removed and the two regions surrounding each gap should be joined, at a point that is referred to as a "split point" through this article. Obviously, a degenerate primer that covers both sequences is located between two split points.

**Figure 8:**
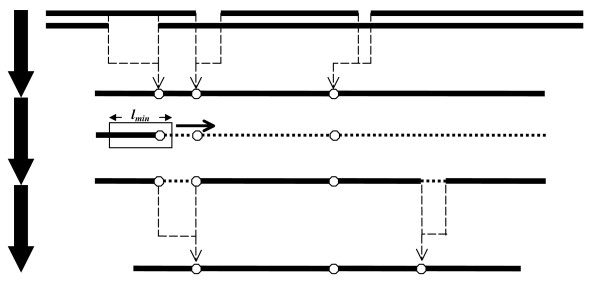
Three steps of merging and refining two aligned sequences in PAMPS: **(1) **Aligned sequences are merged. The regions that occur in a gap are replaced with split points (circles) prior to merging. **(2) **A window of length *l*_*min *_slides through the merged sequence. Once the sequence that occurs within this window possesses degeneracy less than *d*_*max *_and has no split points, all nucleotides of that sequence are marked to be retained (solid lines). **(3) **Regions that their nucleotides are not marked (dotted lines) are replaced with new split points.

After reducing an alignment into a non-gapped, split, degenerate sequence, the regions that in no way can participate in the ultimate degenerate primer should be removed. These regions consist of those having degeneracy larger than *d*_*max *_and those having lengths smaller than *l*_*min*_. To achieve this goal, we only retain those nucleotides that are located within at least one window with length *l*_*min *_and degeneracy at most *d*_*max*_. Obviously, this window cannot have a split point within. If no such a window could be found for a nucleotide, that nucleotide should be removed. This results in the removal of all nucleotides between two split points that are closer than *l*_*min*_. The remaining regions are joined together with a new split point (Figure 8).

### Alignment

The alignment algorithm that is used by PAMPS is very similar to the conventional global alignment [12]. However, the scoring methods differ in some details. Since the purpose is to achieve an alignment that results in a merged sequence with low degeneracy, we defined the score of each match/mismatch as

**Figure 9:**
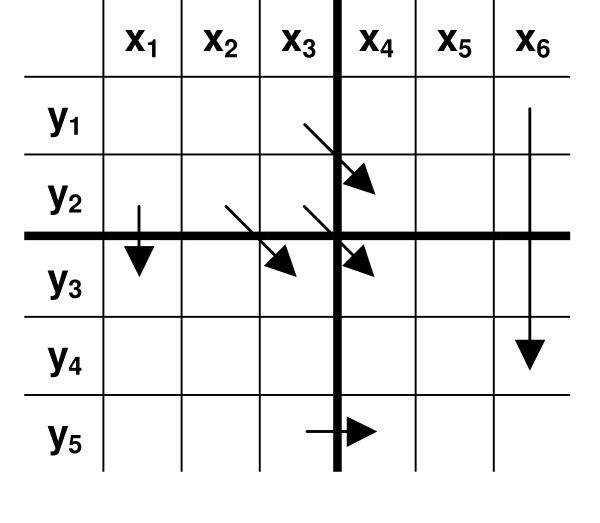
Passing over a split point has the penalty of gap opening. Bold lines indicate the positions of split points in each of the sequences *X *and *Y*. All arrows indicate a score of *g_open*, which is the penalty of gap opening (-10.0 in this work).

(1) *M*(*x*, *y*) = 2 - log_2 _|*x *∪ *y*|.

in which *x*, *y *⊆ ∑. The two sequences that are being aligned may contain some split points as they may themselves have been resulted from merging other sequences. In this case, passing over a split point has the same penalty as gap opening (see Figure 9). In this work, we set the penalty of gap opening to -10.0 and gap extension to 0.0, since for our purpose it is of no importance how long a gap is. Merging two split sequences causes all split points to be copied into the relevant positions in the merged sequence. After two aligned sequences are merged and refined (Figure 8), the alignment score is recalculated, since some of the portions that are scored in the original alignment may be removed in the refined sequence.

### Designing degenerate primers

**Figure 10:**
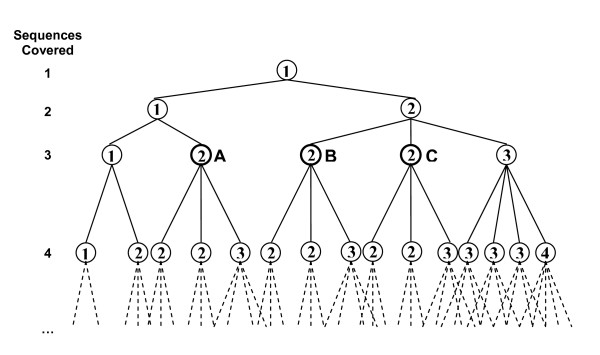
Searching different combinations of sequences to obtain the optimum one. PAMPS starts with the first sequence as *P*_1 _(top node) and either merges the second sequence with it (left branch) or adds the second sequence as *P*_2 _(right branch). The numbers in the nodes represent the number of primers in the corresponding primer sets. Each node is expanded from its left branch first, continuing with the right branches in order. To avoid exponential growth of the tree, some nodes are not expanded. For example, node **B **has the same number of primers as **A **and covers the same number of sequences. Hence, it is expanded only if the sum of scores of its primers exceeds that of **A**. Similarly, **C **is only expanded if it outscores both **A **and **B**.

In order to design degenerate primers, pairs of sequences should be aligned and merged consecutively until no more sequences could be merged (i.e. merging any more pairs of sequences results in primers either with lengths less than *l*_*min *_or with degeneracy more than *d*_*max*_). However, there are different combinations in which sequences can be merged, each of which may result in a different set of primers. The optimum set is the one that contains the least number of primers. PAMPS uses a procedure similar to MIPS [2] to search for the optimum set of primers:

Assume that P = {*P*_1_, *P*_2_, ..., *P*_*m*_} covers the set S = {*S*_1_, *S*_2_, ..., *S*_*n*_} (P covers S if for each *S*_*j *_∈ S, 1 ≤ *j *≤ *n *there is a *P*_*i *_∈ P, 1 ≤ *i *≤ *m *which covers *S*_*j*_). For the *S*_*n*+1 _to be covered by P, *m*+1 "actions" are possible: merging *S*_*n*+1 _with *P*_*i *_(1 ≤ *i *≤ *m*), or adding a new primer (*P*_*m*+1_, which is the same as *S*_*n*+1_) to P. Thus, PAMPS starts with P = {*P*_1_}, *P*_1 _= *S*_1_, and either merges *S*_2 _with *P*_1 _or adds it to P as *P*_2_. P is expanded until it covers all sequences. If in any step one of the requirements of MDPD is not fulfilled, i.e. the length of a primer becomes less that *l*_*min *_or the degeneracy of a primer exceeds *d*_*max*_, PAMPS backtracks to a previous P and continues with another "action" (Figure 10). PAMPS searches for all P's each of which covering all sequences, and chooses the one with the minimum |P| (i.e. chooses the P that covers all sequences with the minimum possible number of primers); however, the minimum |P| is guaranteed only if the following conditions are met; (1) no heuristic approach is employed; (2) no gap is allowed in alignment of sequences, i.e. penalty of gap opening is -8; (3) length of each sequence is equal to required primer length which turns the problem into MP-DPD. Finding the minimum |P| is simply a result of searching all combination of actions, which is obviously not possible for large sets of input sequences; hence the need for a heuristic approach is emerging.

PAMPS uses a similar heuristic approach as MIPS [2] to reduce the search space. Assume P_1 _is a previously found set of primers that contains *m *primers and covers *n *sequences, and P_2 _is a newly found set that also contains *m *primers and covers *n *sequences. P_2 _is only expanded if the sum of scores of its primers (see section Alignment) exceeds that of P_1 _(Figure 10).

## Authors' contributions

HSN developed the algorithm, performed the analysis and participated in preparing the manuscript. NT prepared the background and discussion and drafted the manuscript. MC designed and coordinated the study and contributed in preparing the manuscript. All authors read and approved the final manuscript.

## Supplementary Material

Additional file 1 PAMPS implementation. This compressed package contains the implementation of PAMPS as two Win32 executable files. For more information, please refer to the README.txt that is enclosed within the compressed file. Click here for file
